# Invasive pulmonary aspergillosis often misdiagnosed as lung cancer: A case report

**DOI:** 10.1097/MD.0000000000042705

**Published:** 2025-06-06

**Authors:** Ruiping Bu, Yanhong Zong, Jianping Xu, Jing Yang, Chenda Zhai

**Affiliations:** a Department of Internal Medicine, Graduate School of Hebei Medical University, Shijiazhuang, Hebei, China; b Department of Infectious Diseases, Hebei General Hospital, Shijiazhuang, Hebei, China; c Department of Laboratory Medicine, Hebei General Hospital, Shijiazhuang, Hebei, China; d Department of Internal Medicine, Graduate School of Hebei North University, Zhangjiakou, Hebei, China.

**Keywords:** invasive pulmonary aspergillus, lung cancer, nonimmunocompromised host

## Abstract

**Rationale:**

The incidence of epidemiological pulmonary aspergillosis (PA) is increasing worldwide. Diagnosis of PA is challenging because of the nonspecificity of its clinical manifestations and imaging characteristics. PA has a high mortality rate, making early diagnosis and treatment critical.

**Patient concerns:**

A 67-year-old female patient was admitted to the hospital with a half-month history of cough, blood-tinged sputum, and a sore throat. The patient had no history of chronic diseases, such as hypertension, coronary heart disease, or diabetes. The patient had a family history of cancer.

**Diagnoses:**

The patient was diagnosed with invasive pulmonary aspergillosis (IPA).

**Interventions:**

After the diagnosis of IPA, the patient underwent antifungal treatment with oral voriconazole.

**Outcomes:**

After treatment, the patient’s symptoms improved, and a follow-up chest computed tomography scan showed a reduction in the area of inflammation.

**Lessons:**

Immunocompetent individuals may develop IPA. The clinical and imaging manifestations of IPA vary, which makes misdiagnosis possible. When necessary, a pathological biopsy can be performed to confirm the diagnosis.

## 1. Introduction

Pulmonary aspergillosis (PA) often occurs in immunocompromised and critically ill patients. The most common risk factors are long-term neutropenia, transplantation of hematopoietic stem cells or solid organs, inherited or acquired immunodeficiency, and use of steroids or other immunosuppressants.^[1–3]^ It has a high mortality rate, with an overall 1-year mortality rate of 32%.^[3]^ In rare cases, aspergillosis may occur in individuals with normal immune function.^[4,5]^ The imaging manifestations of invasive pulmonary aspergillosis (IPA) are complex and diverse, among which the typical imaging features of vascular IPA include the air crescent sign, halo sign, and cavity.^[6]^ However, a study by Vandewoude et al^[7]^ showed that typical lesions of invasive aspergillosis, such as halo and crescent signs, were found in only 5% of patients. In this article, we report on a patient with IPA with normal immune function and atypical imaging findings.

## 2. Case presentation

**Figure 1.**
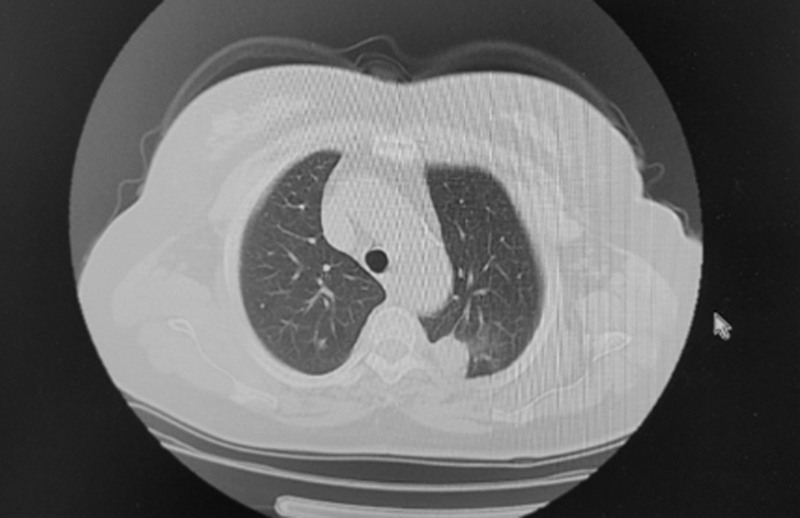
Lung window.

**Figure 2.**
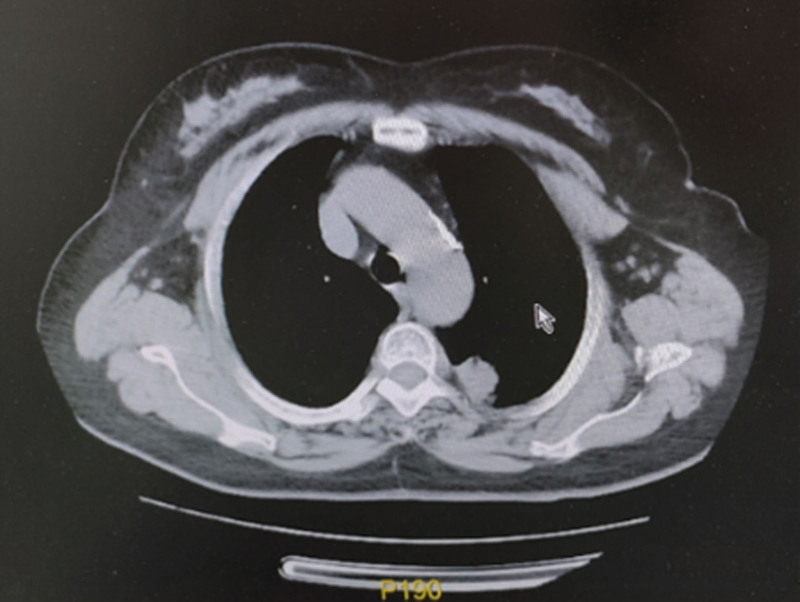
Mediastinal window. Plain chest CT (Figs. 1 and 2) revealed a clustered high-density shadow in the upper lobe of the left lung. CT = computed tomography.

**Figure 3.**
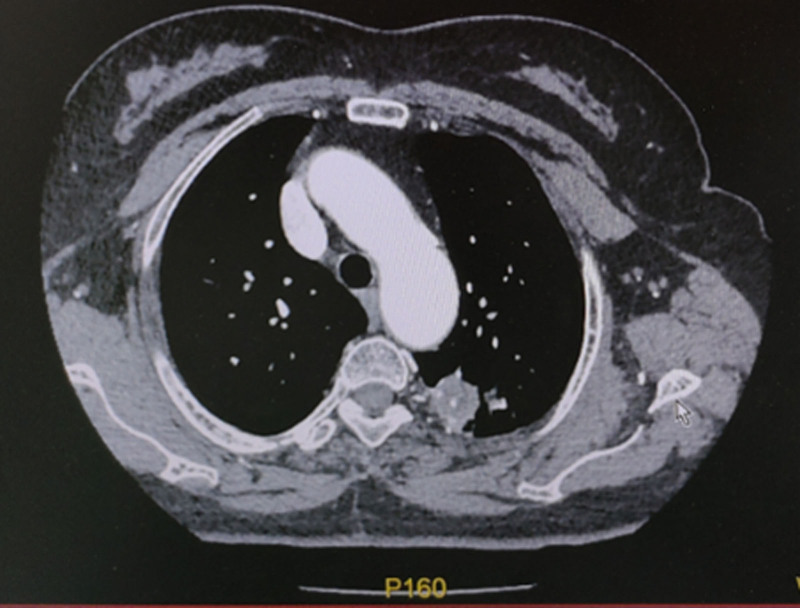
Chest enhanced CT scan. An enhanced scan (Fig. 3) showed uneven enhancement of the upper left lung mass. CT = computed tomography.

The patient was a 67-year-old Han Chinese woman and retired worker from a fertilizer factory. Approximately half a month ago, after cleaning a damp old house, she developed a cough, blood-tinged sputum, and a sore throat. The patient did not experience chest tightness, shortness of breath, fever, or fatigue. After receiving anti-inflammatory treatment at another hospital, the patient’s symptoms did not improve significantly. Plain chest computed tomography (CT) scans (Figs. 1 and 2) showed a clustered high-density shadow in the upper lobe of the left lung, with a maximum area of approximately 31 × 28 mm. Burrs and patchy ground-glass density shadows were observed at the edges of the lesions. An enhanced scan showed uneven enhancement of the upper left lung mass (Fig. 3). The patient had no chronic history of hypertension, coronary heart disease, or diabetes. She had a family history of cancer; her father died of esophageal cancer, her eldest sister had colon cancer, and her second sister died of stomach cancer. Based on the chest CT results from an external hospital and the fact that the patient’s condition did not improve after anti-inflammatory treatment, the preliminary diagnosis was a left lung mass.

**Figure 4.**
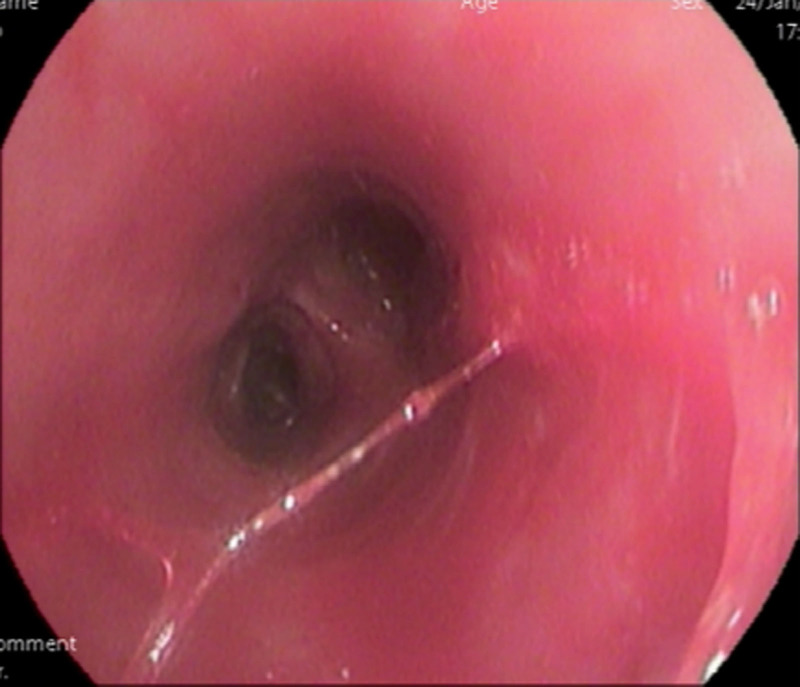
Fiberoptic bronchoscopy shows rough and congested mucosa in the left upper lobe bronchus.

**Figure 5.**
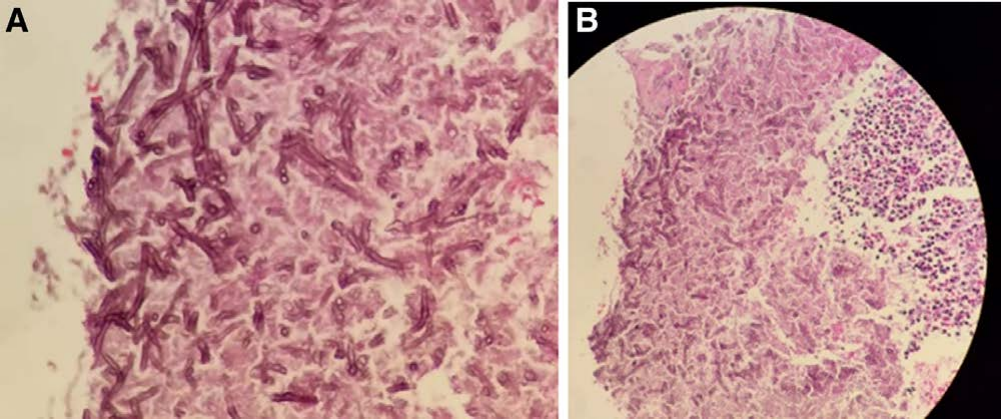
(A) Aspergillus microscopically shows dichotomous acute angle branching septate hyphae. (B) Free Aspergillus clusters were observed in lung puncture tissue.

**Figure 6.**
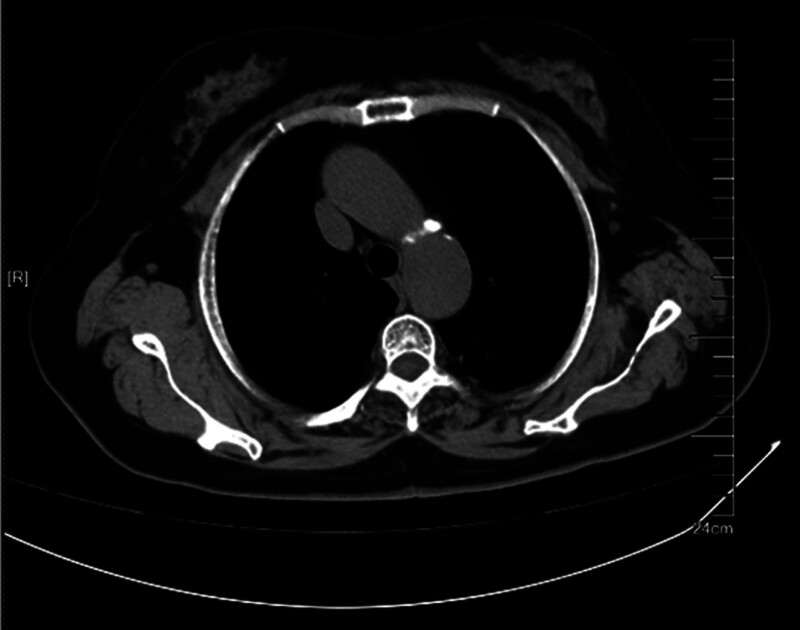
Mediastinal window.

**Figure 7.**
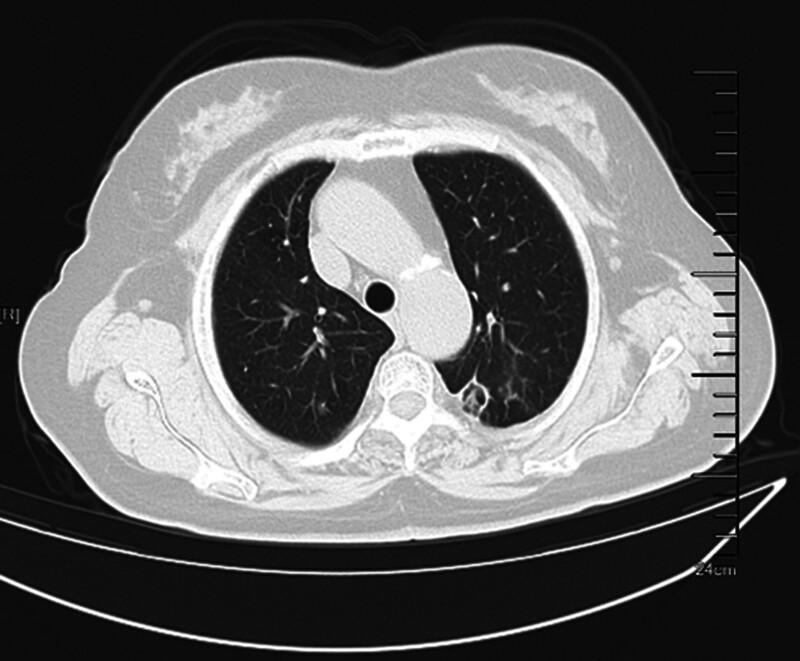
Lung window. A chest CT re-examination 9 days after oral voriconazole treatment (Figs. 6 and 7) showed that the lesion area was reduced. CT = computed tomography.

After admission, the following relevant examinations were completed: routine blood tests, C-reactive protein: 5.31 mg/L, white blood cell count: 8.53 × 10^-9^/L, neutrophils: 6.70 × 10^-9^/L, lymphocytes: 1.38 × 10^-9^/L, Il-6: 3.4 pg/mL, IL-8: 2.5 pg/mL, carcinoembryonic antigen: 6.720 ng/mL, neuronal specific enolase 13.150 ng/mL, and cytokeratin 19 fragment: 3.870 ng/mL. No bacteria or fungi were found in the sputum culture, and serum (1,3)-β-d-glucan (G test) was negative. Fiber bronchoscopy showed (Fig. 4) that the upper left lobe bronchus was open, and the mucosa was rough and congested. The alveolar lavage fluid was collected and sent for bacterial culture and smears, and no pathogenic bacteria were found. The galactomannan (GM) test of bronchoalveolar lavage fluid (BALF) was negative. Lung cancer was not excluded based on patient age, respiratory symptoms, elevated cytokeratin 19 fragments, carcinoembryonic antigen, and imaging findings. Therefore, we performed a CT-guided percutaneous lung biopsy to further define the nature of the lesion. Pathological diagnosis of the lung puncture biopsy showed chronic inflammation in a small amount of bronchial mucosa and a free Aspergillus mass. Microscopically, the Aspergillus showed bifurcated sharp angular branches and isolated mycelia (Fig. 5), resulting in a definitive diagnosis of left PA. The patient was administered oral voriconazole therapy. A chest CT re-examination 9 days after oral voriconazole treatment (Figs. 6 and 7) showed that the lesion area was reduced. The results of the routine blood tests and C-reactive protein levels were normal. No fungus was detected in the sputum smear or culture. The patient was discharged after improvement and oral voriconazole was continued outside the hospital. Chest CT examination was conducted 18 days after discharge; the lesion scope was reduced.

## 3. Discussion

The incidence of IPA has gradually increased with the widespread use of broad-spectrum antibiotics, glucocorticoids, and immunosuppressants. IPA is caused by inhalation of Aspergillus spores and occurs mainly in patients with severe immunosuppression. Spores are ubiquitous in air. These spores come from soil, decomposed plant matter, household dust, building materials, plants and flowers, food and water, etc. The onset of IPA is associated with high spore levels in the environment. Diagnosis of IPA is challenging, and lung histopathology remains the gold standard for IPA diagnosis; however, the fact that many patients do not tolerate tissue biopsy has hindered its widespread clinical use. The diagnosis of IPA is mainly based on clinical manifestations and chest imaging, airway secretion culture, fungal antigen detection (such as GM test), and histopathological examination.^[8]^ IPA is divided into airway and vascular invasive aspergillosis. The main CT findings of airway IPA^[8]^ are as follows: central lobular nodules and tree bud signs are patchy and distributed around the bronchus. The main CT findings of vascular invasive pulmonary Aspergillus disease^[8,9]^ were as follows: early mycelium infiltration of blood vessels formed embolism and bleeding infarct zone, manifested as nodules; the surrounding ground-glass shadow is called the halo sign; and crescent-shaped gas shadow produced by infarcted lung contraction during convalescence. The initial appearance on the patient’s chest CT was clustered high-density imaging, and the chest CT showed a cavity containing filamentous structures. This dynamic change is consistent with the imaging findings of PA; therefore, dynamic observation of chest CT findings is of great value in the diagnosis of IPA.

Clinical manifestations of IPA in patients with normal immune function are atypical. According to a report,^[10]^ the misdiagnosis rate for PA is 73.1%, and it is most frequently misdiagnosed as lung cancer, tuberculosis, or inflammatory pseudotumor. Here, we report a case of IPA with normal immune function, mass density imaging (lesions with uneven enhancement) on CT, and elevated serum cytokeratin 19 fragments. The patient did not have any of the following typical risk factors: long-term immunosuppressant use, blood disease, or diabetes. However, follow-up of the patient’s medical history revealed that the patient had cleaned a dark and damp old house before the onset of the disease. The high concentration of Aspergillus in the dark and humid environment may be the cause of the patient’s illness. When patients inhale high concentrations of Aspergillus spores, drown, or fall into a fecal pool, even those with normal immune function can develop IPA.^[5,11–13]^ Therefore, inquiring about a patient’s history, especially the patient’s living environment, can help diagnose IPA.

GM is an important index for the diagnosis of IPA. de Oliveira et al^[14]^ showed that the sensitivity and specificity of bronchoalveolar lavage fluid GM for IPA diagnosis were 0.68 and 0.84. BALF GM performed better than serum. As the amount of GM released is proportional to the amount of bacteria, the GM value can also reflect the degree of infection. In this case, the patient’s GM test was a false negative, which may be related to the patient’s use of antibiotics in other hospitals. The use of antibiotics can inhibit the release of GM, thereby reducing the positive rate of GM testing. Therefore, a single serum and/or BALF GM antigen test is insufficient, and the diagnosis of IPA should be evaluated in combination with mycological and pathological factors.

The patient had normal immune function, a space-occupying lesion on chest CT, and elevated serum tumor markers, which misled our diagnosis. The detection of serum tumor markers has high specificity, among which carcinoembryonic antigen and cytokeratin 19 fragment are 2 commonly used tumor markers related to lung cancer. Cytokeratin 19 fragment detection has high diagnostic value in non–small cell lung cancer, especially in lung squamous cell carcinoma patients,^[15]^ but its sensitivity is poor.^[16]^ Therefore, this case warns us that when a patient’s chest image is lumpy with high-density shadows, accompanied by elevated serum tumor markers, we should not limit the diagnosis to lung cancer, but also consider the possibility of infectious disease. Overall, this case adds to the literature on atypical imaging manifestations of IPA and highlights the importance of considering fungal infections in the differential diagnosis of respiratory diseases.

## 4. Conclusion

This case shows that individuals with normal immune function may also suffer from IPA. When patients present with cough, hemoptysis, fever, and other symptoms, even if the patient has no risk factors for IPA and chest CT has no typical imaging features of aspergillosis, inquiring about the patient’s contact environment in detail is helpful for the differential diagnosis of the disease, and lung tissue biopsy should be actively performed to confirm the diagnosis.

## Author contributions

**Project administration:** Jing Yang.

**Supervision:** Jing Yang.

**Writing – review & editing:** Ruiping Bu, Jing Yang, Yanhong Zong.

**Conceptualization:** Ruiping Bu, Yanhong Zong, Chenda Zhai.

**Formal analysis:** Ruiping Bu, Jianping Xu.

**Investigation:** Ruiping Bu, Yanhong Zong, Chenda Zhai.

**Writing – original draft:** Ruiping Bu.

**Data curation:** Yanhong Zong, Jianping Xu, Chenda Zhai.

**Resources:** Ruiping Bu, Yanhong Zong.

## References

[1] Machado M Fortún J Muñoz P. Invasive aspergillosis: a comprehensive review. Med Clin. 2024;163:189–98. 10.1016/j.medcli.2024.01.045 38714471

[2] Kosmidis C Denning DW. The clinical spectrum of pulmonary aspergillosis. Thorax. 2014;70:270–7. 25354514 10.1136/thoraxjnl-2014-206291

[3] Henao-Martínez AF Corbisiero MF Salter I Chastain DB Thompson GR. Invasive pulmonary aspergillosis real-world outcomes: clinical features and risk factors associated with increased mortality. Med Mycol. 2023;61:myad074. 37491703 10.1093/mmy/myad074

[4] Sethi SM Arshad A. An immunocompetent lady with invasive aspergillosis presenting as disseminated lesions: a case report. J Med Case Rep. 2024;18:354. 39103930 10.1186/s13256-024-04579-z PMC11301935

[5] Ao W Huang P Wang J Fu X Fu B. Fatal invasive pulmonary aspergillosis in non-immunocompromised host: a case report. Medicine (Baltimore). 2023;102:e35702. 37904478 10.1097/MD.0000000000035702 PMC10615459

[6] Moldoveanu B Gearhart AM Jalil BA Saad M Guardiola JJ. Pulmonary aspergillosis: spectrum of disease. Am J Med Sci. 2020;361:411–9. 33563417 10.1016/j.amjms.2020.12.009

[7] Vandewoude KH Blot SI Depuydt P. Clinical relevance of Aspergillus isolation from respiratory tract samples in critically ill patients. Crit Care. 2006;10:R31. 16507158 10.1186/cc4823 PMC1550813

[8] Ullmann AJ Aguado JM Arikan-Akdagli S. Diagnosis and management of Aspergillus diseases: executive summary of the 2017 ESCMID-ECMM-ERS guideline. Clin Microbiol Infect. 2018;24(Suppl 1):e1–e38. 29544767 10.1016/j.cmi.2018.01.002

[9] Ledoux MP Guffroy B Nivoix Y Simand C Herbrecht R. Invasive pulmonary aspergillosis. Semin Respir Crit Care Med. 2020;41:80–98. 32000286 10.1055/s-0039-3401990

[10] Zhang R Wang S Lu H Wang Z Xu X. Misdiagnosis of invasive pulmonary aspergillosis: a clinical analysis of 26 immunocompetent patients. Int J Clin Exp Med. 2014;7:5075–82. 25664007 PMC4307454

[11] Rengifo LM Haywood JD Koberlein GC Zeller KA Ramirez KA. Invasive pulmonary aspergillosis in a 4-year-old male following submersion in a manure pond. Future Microbiol. 2023;18:933–8. 37650709 10.2217/fmb-2023-0018

[12] Tavazzi G Via G Marzani FC Mojoli F. Invasive pulmonary aspergillosis after near-drowning. Lancet Infect Dis. 2016;16:1430. 27998605 10.1016/S1473-3099(16)30202-X

[13] Koide S Hadano Y Mizuochi S Koga H Yamashita H. Invasive aspergillosis after non-fatal drowning. Int Med Case Rep J. 2020;13:77–83. 32210640 10.2147/IMCRJ.S241234 PMC7069574

[14] de Oliveira VF Silva GD Taborda M Levin AS Magri MMC. Systematic review and meta-analysis of galactomannan antigen testing in serum and bronchoalveolar lavage for the diagnosis of chronic pulmonary aspergillosis: defining a cutoff. Eur J Clin Microbiol Infect Dis. 2023;42:1047–54. 37430166 10.1007/s10096-023-04639-0

[15] Fu L Wang R Yin L Shang X Zhang R Zhang P. CYFRA21-1 tests in the diagnosis of non-small cell lung cancer: a meta-analysis. Int J Biol Markers. 2019;34:251–61. 31436122 10.1177/1724600819868234

[16] Yang Q Zhang P Wu R Lu K Zhou H. Identifying the best marker combination in CEA, CA125, CY211, NSE, and SCC for lung cancer screening by combining ROC curve and logistic regression analyses: is it feasible? Dis Markers. 2018;2018:2082840. 30364165 10.1155/2018/2082840 PMC6188592

